# *FANSe2*: A Robust and Cost-Efficient Alignment Tool for Quantitative Next-Generation Sequencing Applications

**DOI:** 10.1371/journal.pone.0094250

**Published:** 2014-04-17

**Authors:** Chuan-Le Xiao, Zhi-Biao Mai, Xin-Lei Lian, Jia-Yong Zhong, Jing-jie Jin, Qing-Yu He, Gong Zhang

**Affiliations:** Key Laboratory of Functional Protein Research of Guangdong Higher Education Institutes, Institute of Life and Health Engineering, College of Life Science and Technology, Jinan University, Guangzhou, China; Beijing Institute of Genomics, Chinese Academy of Sciences, China

## Abstract

Correct and bias-free interpretation of the deep sequencing data is inevitably dependent on the complete mapping of all mappable reads to the reference sequence, especially for quantitative RNA-seq applications. Seed-based algorithms are generally slow but robust, while Burrows-Wheeler Transform (BWT) based algorithms are fast but less robust. To have both advantages, we developed an algorithm FANSe2 with iterative mapping strategy based on the statistics of real-world sequencing error distribution to substantially accelerate the mapping without compromising the accuracy. Its sensitivity and accuracy are higher than the BWT-based algorithms in the tests using both prokaryotic and eukaryotic sequencing datasets. The gene identification results of FANSe2 is experimentally validated, while the previous algorithms have false positives and false negatives. FANSe2 showed remarkably better consistency to the microarray than most other algorithms in terms of gene expression quantifications. We implemented a scalable and almost maintenance-free parallelization method that can utilize the computational power of multiple office computers, a novel feature not present in any other mainstream algorithm. With three normal office computers, we demonstrated that FANSe2 mapped an RNA-seq dataset generated from an entire Illunima HiSeq 2000 flowcell (8 lanes, 608 M reads) to masked human genome within 4.1 hours with higher sensitivity than Bowtie/Bowtie2. FANSe2 thus provides robust accuracy, full indel sensitivity, fast speed, versatile compatibility and economical computational utilization, making it a useful and practical tool for deep sequencing applications. FANSe2 is freely available at http://bioinformatics.jnu.edu.cn/software/fanse2/.

## Introduction

Mapping (aligning) millions of next-generation sequencing (NGS) reads accurately to reference sequences is the basis of all deep sequencing applications that utilize reference genomes or transcriptomes, including variant analysis, gene expression and isoform analysis. Traditional alignment algorithms such as BLAST and BLAT could not process the massive amount of sequencing data in hours (reviewed in [1]). A series of early mapping algorithms such as SSAHA, MAQ and SOAP started to tackle this speed hindrance. These algorithms extended the basic idea of “seeding” (hash table indexing) from BLAST, which is simple in design and easy to implement, bringing the NGS technology into quantitative era (reviewed in [2], [3]). The computational time of this type of algorithms is theoretically proportional to the size of reference sequence ([4] and reviewed in [2]). Therefore accurately mapping to large genomes is still time-consuming [5], [6]. Another type of algorithms based on Burrows-Wheeler Trasnformation (BWT), e.g. Bowtie and BWA, takes the advantage of the suffix/prefix trie and thus reduces the computational complexity, being typically 5∼20x faster than seed-based algorithms (reviewed in [2], [7]). Such methods can map tens of millions of reads to human genome within one day on desktop workstations, thus promoting the blowout of NGS applications. According to a statistics till the end of 2012, two among the top three cited mapping algorithms are of this type (Bowtie and BWA) (reviewed in [6]). In real-world benchmarks, although the sensitivity of earlier BWT-based algorithms like Bowtie and SOAP2 (<80%) is still to be improved when mapping DNA sequencing reads, the sensitivity of the upgraded Bowtie2 is almost the same as the traditional seed-based algorithms while being more than 20x faster [6].

However, deviations between reads and reference sequences set a great challenge of the sensitivity and speed to the mapping algorithms. Origins of the deviation include single nucleotide polymorphisms (reviewed in [8]), PCR amplification [9], base calling (reviewed in [10]) and sequencer errors [11]. When the mismatch rate exceeds 2% or the indel rate exceeds 0.5%, most algorithms lose their accuracy [12]. Due to the principle of BWT, this type of algorithms is less error-tolerant and thus usually less sensitive than seed-based algorithms at higher error rate (reviewed in [1], [7]). For RNA-seq, the error rate is higher due to RNA editing [13], modifications [14] and nucleotide misincorporation in reverse transcription [15]. Indeed, in simulated tests, Bowtie and BWA remained 55%∼75% accuracy at 4% error rate, while the seed-based algorithms SOAP and Novoalign maintain 80%∼90% accuracy [12]. This result coincides with the real-world test: even when adding the splice-mapped reads, BWT-based algorithms TopHat and SOAPsplice mapped 12∼19% of reads less than seed-based algorithms [6]. These algorithms tend to unproportionally lose mappable reads of the medium to low abundance RNA, generating a significant bias in quantification [5]. Moreover, the accuracy of BWT-based algorithms was shown to be highly dependent on the dataset in various comparative tests, from very high [7] to moderate [6], [12] to very low [16], in contrast to the seed-based algorithms. The inconsistency of quantitative results given by RNA-seq and microarray may reflect this bias and unrobustness [17]–[19].

It would be ideal to combine the speed of BWT and the robust accuracy of seed-based algorithms, especially for the cases with higher error rates like RNA-seq. To improve the robustness and indel detection of the BWT-based algorithm Bowtie, the upgraded Bowtie2 partially took the advantage of the seeding principle, and it truly exceeded Bowtie, BWA and SOAP2 [20]. However its accuracy and robustness are difficult to be theoretically estimated. To overcome this problem, we took the advantage of our previously developed FANSe algorithm, which is a seed-based algorithm with theoretical estimation of high accuracy and robustness (miss rate can be as low as 10^−6^) [5], and further developed FANSe2 algorithm. FANSe2 can map a billion reads to human genome in hours using normal office computers without compromising the high and robust accuracy. We also tested this algorithm using real-world RNA-seq datasets and experimentally validated its results by RT-PCR and microarray.

## Materials and Methods

### Design of FANSe2

**Figure 1:**
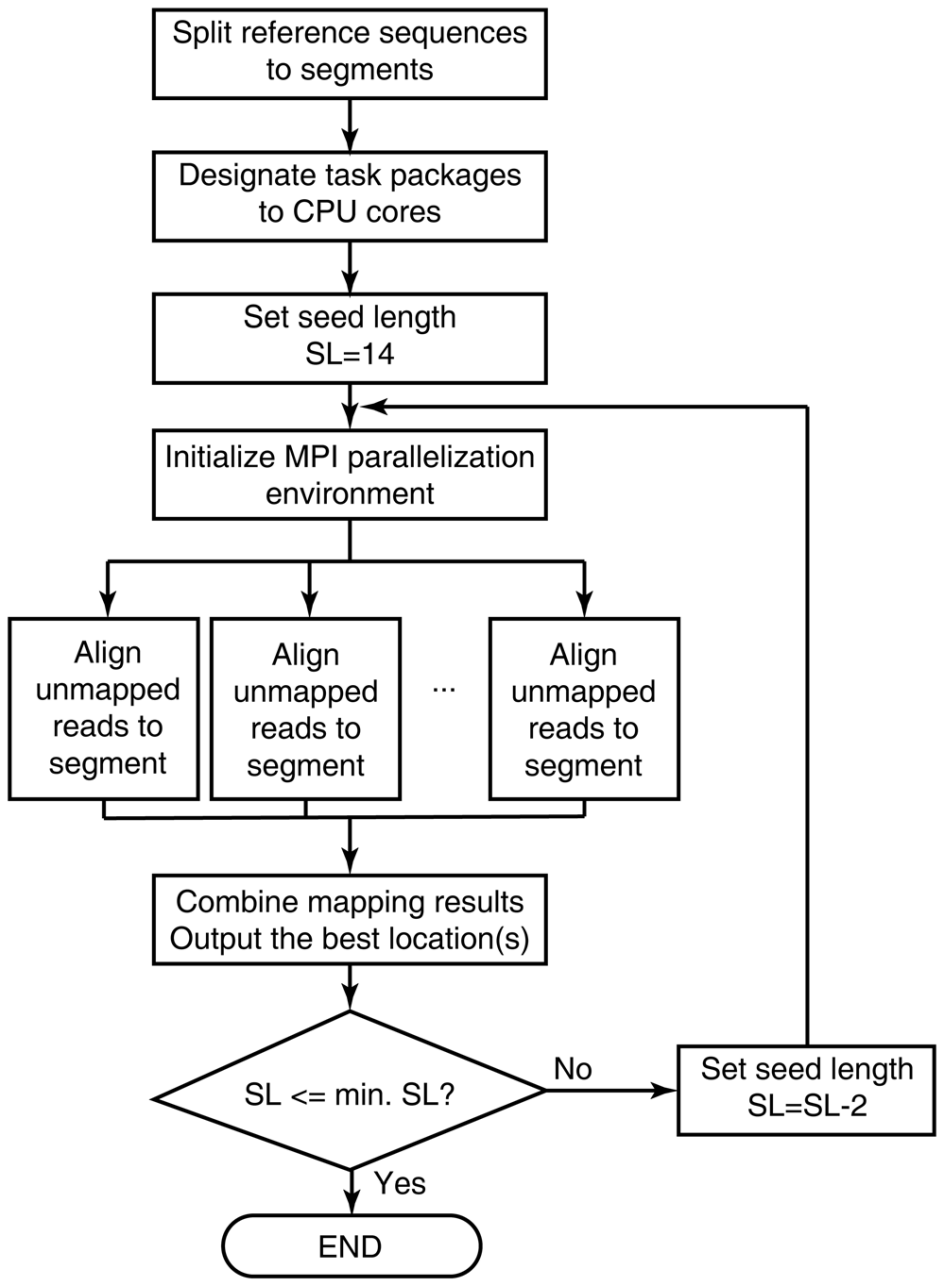
Flowchart of FANSe2. For details please refer to the Materials and Methods section. SL  =  seed length.

FANSe2 is an iterative and parallel seed-based read mapping algorithm with a simple design to ensure all advantages of FANSe and largely improve the speed and parallelization. The following major steps were implemented: (Figure 1).

#### Step 1

Segmentation of reference sequences. To reduce the memory consumption, large reference sequences like human genome are split to segments. Two adjacent segments are overlapped with maximum read-length. Each segment will be processed as a task package and assigned to a processor core.

#### Step 2

Initialize parallel computing environment. To avoid resource competition, FANSe2 parallelizes multiple processes via the industrial standard MPICH2 environment instead of multi-threading. Unlike FANSe that uses the 6- or 8-nt seeds, FANSe2 initially set the seed length as 14-nt.

#### Step 3

Each CPU core starts to process the assigned task package, mapping all reads to the reference sequence segment using the seed length based on the principle of FANSe. The final refinement of hotspots is performed by calculating Hamming distance (indel detection off) or by using accelerated Smith-Waterman method (indel detection on) [5].

#### Step 4

After all the task packages are processed, the mapping results are combined and the best mapping location of a read is written to the final result file.

#### Step 5

FANSe2 decreases the seed length by 2-nt and tries to map the unmapped reads using the shorter seed length (back to step 3). Iterative mapping process stops when the seed length reaches the minimum seed length or all the reads are mapped.

### Datasets and reference sequences

To analyze the nucleotide error distribution in the sequencing datasets, we downloaded six datasets from DDBJ Sequence Read Archive (http://trace.ddbj.nig.ac.jp/dra/index_e.shtml), as listed in Table S1 in File S1. Each read was truncated at the nucleotide, whose sequencing quality is lower than 20 in Phred scale. Reads shorter than 18 nt were discarded. The *E. coli* datasets were mapped to *E. coli* K-12 substrain MG1655 genome sequence (NCBI Reference Sequence: NC_000913.2). The yeast datasets were mapped to *S. cerevisiae* genome sequence sacCer3 (downloaded from UCSC genome browser, http://hgdownload.cse.ucsc.edu). FANSe was used to perform these mappings with the errors allowed as listed in Table S1 in File S1 and indel detection on.

The *E. coli* mRNA dataset reported previously was used to test the sensitivity and speed of FANSe2 [5]. The datasets of the whole Flowcell A (FCA) of Human Body Map 2.0 project, containing altogether 608 million 75-nt reads of human polyA^+^ mRNA sequenced on an Illumina HiSeq-2000 sequencer, were used to test the parallel computing capacity of FANSe2. The human datasets were mapped to human genome sequence hg19/GRCh37 (downloaded from UCSC genome browser).

Simulated datasets with 2% and 4% error rate were generated from human chromosome 1 non-masked and masked genome sequence (hg19/GRCh37). Each datasets contained 500,000 reads, 75-nt long. These reads were generated from the non-masked regions. These datasets were mapped to human chromosome 1 non-masked and masked genome sequence, respectively. Reads with homopolymeric stretch or dinucleotide repeats longer than half of the read length were filtered out to avoid unnecessary and ambiguous alignment [21].

### RT-PCR validation of mapping results for RNA-seq

We previously sequenced the total RNA of lung adenocarcinoma A549 cells and sequenced poly-A+ mRNA [22]. The reads were mapped to human mRNA reference sequence (RefSeq) for GRCh37/hg19 (downloaded from UCSC genome browser, accessed on Jan. 21, 2013) using both FANSe2 and Bowtie2. The parameters for FANSe2 was –E7 –I1 –S12, and the parameters for Bowtie2 was —very-sensitive. The mRNAs were quantified using standard rpkM method [3]. Genes with less than 10 reads mapped were considered as unreliable quantified genes and removed [23].

This total RNA sample was reverse transcribed with poly-dT primer using RevertAid Premium reverse transcriptase (Fermentas) and specific genes were amplified using specific primers. PCR was performed using gene-specific primers (Table S2 in File S1) and DreamTaq Green Mix (Fermentas) enzyme. We used the primers in the Whole Transcriptome qPCR Primers Database if available [24], otherwise we used the online tool NCBI PrimerBLAST (http://www.ncbi.nlm.nih.gov/tools/primer-blast/) to design gene-specific primers automatically. The PCR cycle was set as 95°C denaturing for 30 seconds, 59°C annealing for 30 seconds, and 72°C elongation for 30 seconds (amplicon size <500 bp) or 1.2 minutes (amplicon size 500∼1200 bp). 35 PCR cycles were conducted for each reaction. The PCR products were resolved on 2.7∼3% agarose gels and visualized by SybrGreen staining.

### Comparison of NGS and microarray quantifications

The Affymetrix Rat Genome 230 2.0 microarray dataset and Illumina GAIIx RNA-seq dataset of the aristolochic acids treated rat liver sample AA_1 from a previous study [25] was downloaded from Gene Expression Omnibus (GEO) database (accession numbers: GSE5350 and GSE21210). The normalization of the microarray data was performed using RMA method as previously described [25], [26]. In case that multiple probe sets were present for a gene, the probe set with the highest signal intensity was used for this gene [27]. The RNA-seq quantification result using Bowtie was downloaded from GSE21210. We mapped the original RNA-seq using FANSe2 to the reference transcriptome sequence RefSeq release 47 as mentioned in that study [25] with the options –L36 –E3 –S10. The splice variants were merged. RNA-seq quantifications were based on rpkM method [3].

### Comparison of mapping programs

We compared FANSe2 with FANSe, Bowtie, Bowtie2, BWA, SHRiMP2 and Novoalign (for details please refer to Table S3 in File S1). The performance tests were carried out on quad-core Intel i5-3570K computers with 16 GB RAM. We used –n 3 —tryhard—best for Bowtie and –n 7 –o 1 for BWA. Unless specified, —very-sensitive option was used for all Bowtie2 tests. The memory consumption of these programs was recorded using either Task Manager (Windows) or System Monitor (Linux).

Sensitivity and correctness were defined previously [5]. In brief, a read that is truly originated from the reference sequence can have one of the following three outcomes after being processed by an algorithm: (i) mapped to its correct position (*C*); (ii) mapped to a wrong position (*I*); (iii) failed to be mapped to the reference genome (*U*). Sensitivity is defined as 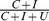, and the correctness is defined as 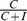. Sensitivity can be calculated from a deep-sequencing dataset, which is proportional to the number of mapped reads. Correctness can be only evaluated using simulated random datasets.

## Results

### Iterative step-down acceleration strategy based on the real-world alignment error distribution

**Figure 2:**
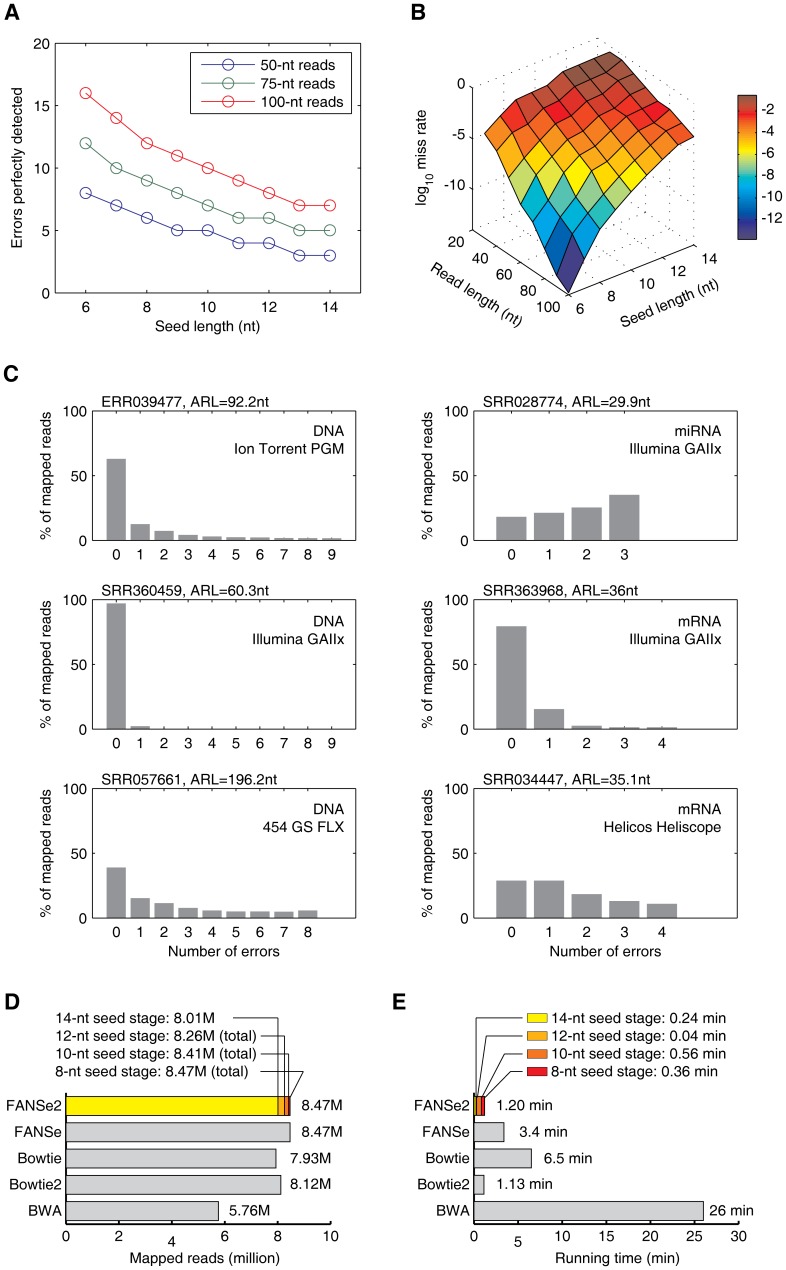
Rational and validation of the iterative strategy of FANSe2. (A) Errors in a read which can be perfectly detected by FANSe algorithm versus the seed length for 50-, 75- and 100-nt reads, respectively. (B) Theoretical miss rate of FANSe algorithm with different seed length for various read length. (C) Error distribution of six sequencing datasets (listed in Table S1 in File S1) sequenced on various types of sequencers, respectively. Reads were mapped with FANSe. ARL  =  average read length. (D, E) Mapping the *E. coli* mRNA dataset reported in [5]. For FANSe2 algorithm, the reads mapped (D) and the calculation time (E) used using different read length stages were shown in colors. The test was performed in a quad-core Intel i5-3570K computer using one CPU core.

When mapping a read, FANSe takes seeds (6- or 8-nt long) from the read and searches for exact matches in the reference genome with a pre-built look-up table [5]. These exact matches are then merged into hotspots and then refined to determine the best mapping location. An *n*-nt long seed has in average *N*/4*^n^* exact matches in the genome (where *N* is the genome size), a large number for large genomes and *n* = 8, thus creating a heavy workload for the hotspot merging and refinement, especially when indel detection is enabled. Longer seeds decrease the number of exact matches exponentially and thus largely accelerate the mapping: 14-nt seed decreases the number of exact matches 4^14–8^ = 4096 folds than 8-nt seeds. However, longer seeds are more likely to contain error and may lose the reads with higher number of mismatches, thus impairing the sensitivity. A read containing maximum *f* errors with a minimal read length of *n*(*f*+1) can be reliably mapped to a genome when using *n*-nt seeds, indicating that a long read with a few errors may be still stably mapped with longer seeds (Figure 2A). For example, up to 5 errors are guaranteed to be detected in 75-nt reads using 14-nt seeds. To achieve theoretical miss rate less than 1%, 12-nt seeds are sufficient for 50-nt reads, whereas 14-nt seeds are more than enough for 100-nt reads (Figure 2B). Decreasing the seed length to 10-nt may reach the theoretical miss rate 10^−4^∼10^−8^.

We then analyzed the actual error distribution in real-world datasets. We mapped six datasets including DNA-seq, mRNA-seq and miRNA-seq datasets obtained from various sequencing platforms using FANSe algorithm (Table S1 in File S1). Notably, a large fraction of the mappable reads contained very few errors, regardless in DNA-seq or RNA-seq datasets (Figure 2C). More than half of the mappable reads contain 0 or 1 error in most cases, and they can be reliably mapped with 14-nt seeds in much higher speed. Therefore, we implemented an iterative step-down strategy: long seeds (e.g. 14-nt or 12-nt) are used to map most reads with high speed, and the unmapped reads (a small fraction) are mapped in the next iteration with shorter seed. This iterative process terminates when the seed length reaches the limit set by the user (Figure 1).

We tested this strategy with the *E. coli* mRNA-seq dataset that was previously used in FANSe test [5]. Stepping down to 8-nt seed length, FANSe2 exported the same mapping result as FANSe at much faster speed using single CPU core when allowing 3 mismatches (Figure 2D and 2E). Indeed, most of the mappable reads were mapped in the initial iteration using 14-nt seeds. When stepped down to 12-nt seeds, FANSe2 mapped 8.26 M reads using 0.28 minutes in total. At this stage, the sensitivity is already higher than the widely-used Bowtie and Bowtie2 (7.93 M and 8.12 M reads, respectively, Figure 2D), while faster than Bowtie2 (1.13 minutes). Stepping further down to 8-nt stage may not be practically necessary, since this significantly increased the running time for three times, however only mapped 0.21 M more reads. Even down to 8-nt stage, the speed of FANSe2 is 3∼21x faster than FANSe, Bowtie and BWA, only slightly slower than Bowtie2 (Figure 2E).

### Memory consumption, speed and scalability when handling huge datasets

**Figure 3:**
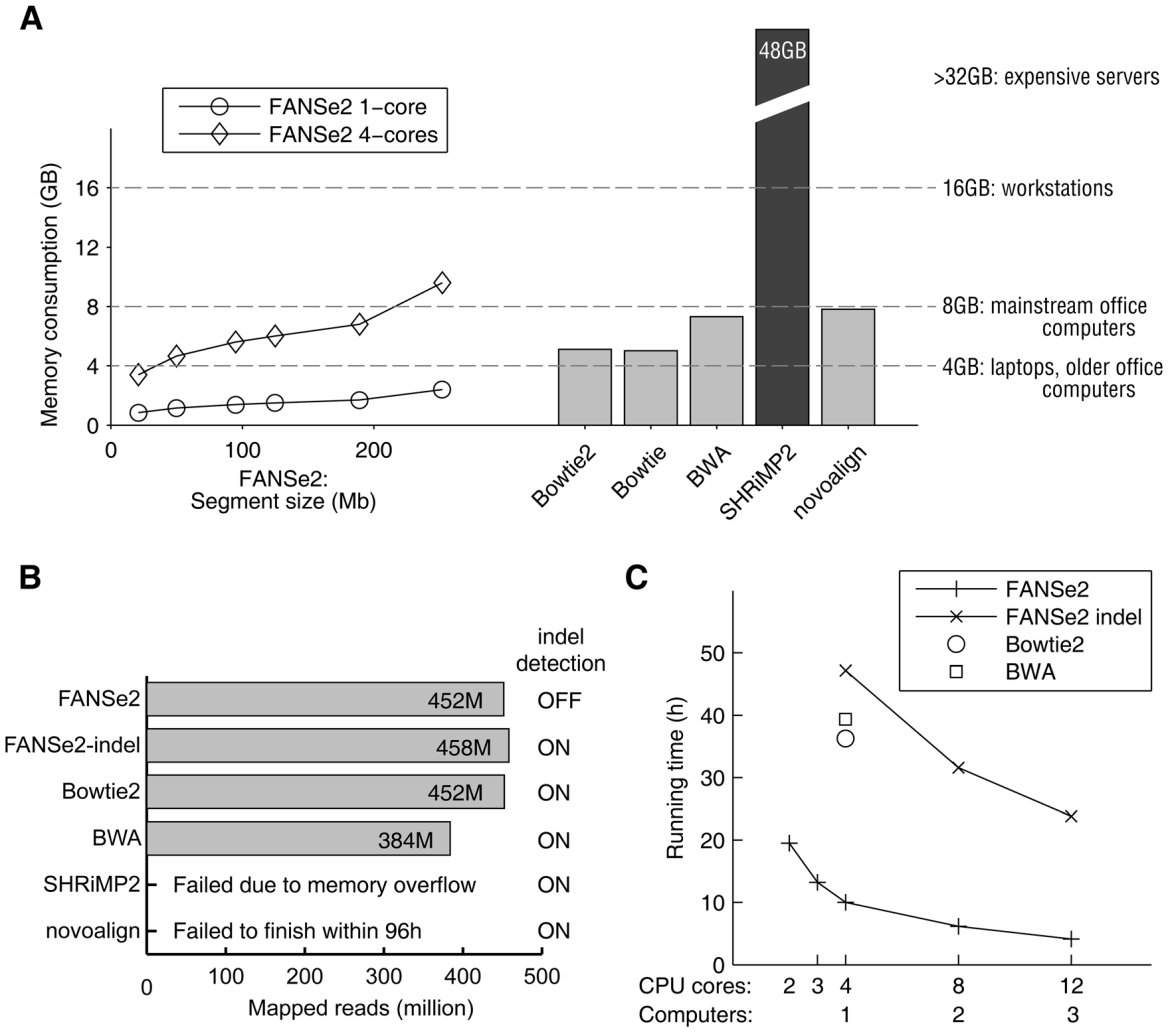
Scalability, sensitivity and speed of FANSe2 compared to other algorithms. (A) Memory consumption of the tested algorithms when mapping 75-nt reads to human genome. The memory consumption of FANSe2 using 1 CPU core and 4 CPU cores are indicated using circles and diamonds, respectively. SHRiMP2 failed to run this test in our 16 GB memory system; thus its memory consumption was taken from its manual. (B, C) Mapping data from an entire Illumina HiSeq-2000 flowcell (608 M 75-nt reads) to masked human genome. (B) The number of reads mapped by the tested algorithms using one computer (4 CPU cores). FANSe2 was tested with indel detection on and off, respectively. SHRiMP2 failed to run in our system due to its high memory consumption. Novoalign failed to finish the task within 96 hours. (C) The time to perform this mapping using different number of CPU cores and computers. Plus sign: FANSe2 without indel detection; cross: FANSe2 with indel detection. Bowtie2 (circle) and BWA (rectangle) do not support automatic parallelization across multiple computers.

The memory consumption of FANSe2 is tunable by the user, because it is only relevant to the genome segment size: FANSe2 uses 1.2∼1.7 GB memory for each activated CPU core when the reference sequences are split to 50∼200 Mb segments (Figure 3A). Therefore, FANSe2 can accelerate mapping large datasets by using 2∼4 CPU cores on one computer using 4∼8 GB RAM. This means that even laptop computers can perform mapping with ease. When mapping reads to human genome, Bowtie2 and Bowtie needs more than 5 GB RAM. BWA and novoalign needs 7.3∼7.8 GB RAM, which hardly fits a computer with 8 GB RAM because the operating system usually requires additional 0.7∼1.2 GB RAM (Figure 3A). According to the manual, SHRiMP2 needs 48 GB RAM to map reads to human genome, which is already far beyond the capacity of high-end workstations, including our computers (Figure 3A and 3B).

In addition, FANSe can parallelize across multiple normal computers with simple LAN connection, providing a economic and scalable solution for biology labs. This feature is not offered by any other current mainstream mapping tools. We tested the scalability of FANSe2 in our real office environment with three heterologous computers: two Intel i5-3570s and one Intel i5-2500 with 8 GB∼16 GB RAM installed, connected with gigabit LAN. One such inexpensive office computer (∼$600) mapped an mRNA-seq dataset of Human Body Map 2 generated from an entire Illumina HiSeq-2000 flowcell (8 lanes, 608 M reads, 75-nt) to human reference genome within 10 hours, 3.6x faster than Bowtie2 in very sensitive mode while maintaining the same sensitivity. With three computers and one-click run, the same job finished 4.1 hours by FANSe2 (Figure 3B and 3C). With the indel detection enabled, FANSe2 mapped these reads within 23.8 hours with three computers. FANSe2 with indel search mapped more reads than Bowtie2 and BWA, which were also enabled indel search (Figure 3B). Compared with one computer, three computers accelerated the mapping for 2.43x (Figure 3C). Note that this efficient parallelization was performed with user-friendly graphical user interface. In contrast, SHRiMP2 failed to run because of its high memory demand. Novoalign was unable to finish the task in 4 days (Figure 3B). These results showed that FANSe2, as a seed-based algorithm, is approaching the speed of BWT-based algorithms while maintaining similar or higher sensitivity when handling huge datasets.

### Sensitivity and correctness of FANSe2 tested with simulated dataset

**Figure 4:**
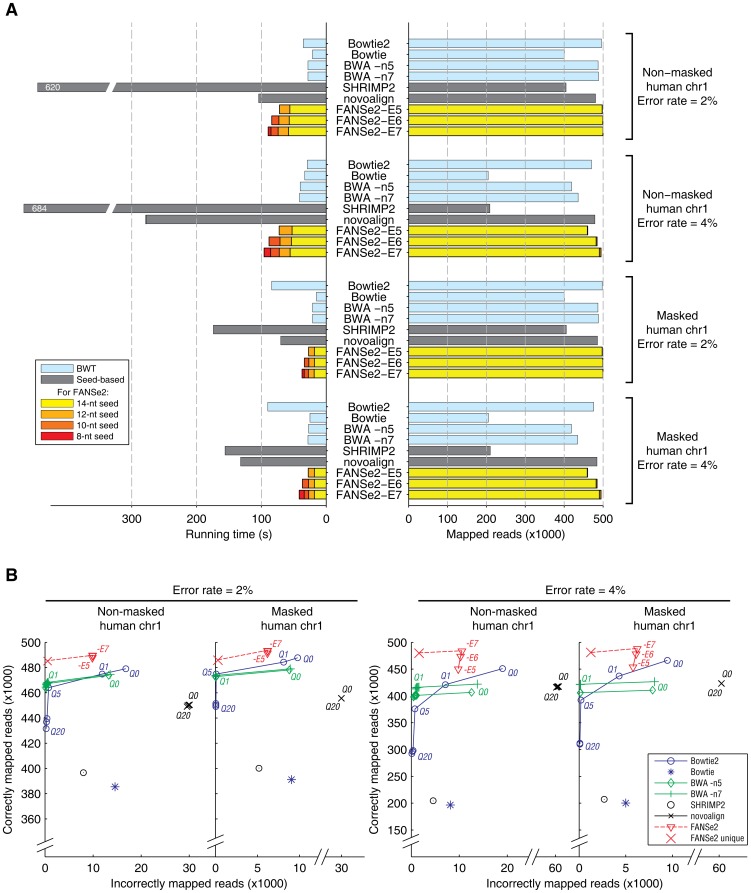
Comparison of FANSe2 and other algorithms on their sensitivity, speed and correctness using simulated datasets from non-masked and masked human chromosome 1 reference sequence (hg19) with 2% and 4% sequencing error rate, respectively. Each dataset contains 500,000 reads with the length of 75-nt. The test parameters are listed in Table S4 in File S1. (A) Comparative test on sensitivity and speed. Reads mapped and the time used at different stages of seed lengths in FANSe2 are shown in colors. The BWT-based algorithms are shown in light blue bars, and the other seed-based algorithms are shown in gray bars. (B) Comparative test on correctness. For Bowtie2, BWA and novoalign, mapped reads were filtered using various mapping quality threshold (Q0∼Q20) represented in Phred score scale (black circle). The correctness of FANSe2 results were marked on the same plot when considering all mapping results (red triangle, 5∼7 errors allowed) or considering only the reads that were uniquely mapped (red cross, 7 errors allowed). The results of Bowtie and SHRiMP2 were not filtered according to the mapping quality due to their low mapping sensitivity.

Practically, the raw error rates of the current next-generation sequencing platforms were reported as 0.26∼13% [11], and the base calling step adds further 2.76∼4.86% error rate [10]. Therefore, a mapping algorithm should reliably map reads containing at least such errors. To test the sensitivity and correctness of FANSe2 algorithm, we generated four simulated datasets, each containing 500,000 reads of 75-nt Illumina-like single-end reads, from the non-masked and masked human chromosome 1 genome sequence (hg19) and with substitution rate of 2% and 4%, respectively. For all four cases, the speed of traditional BWT-based algorithms (Bowtie, Bowtie2 and BWA) are generally faster than traditional seed-based algorithms (SHRiMP2 and novoalign). This coincides with the previous comparisons [6]. However, FANSe2 is just slightly slower than BWT-based algorithms in all cases, and is even faster than Bowtie2 when using the masked genome. In all four cases, FANSe2 mapped more reads than all other tested algorithms (Figure 4A). The sensitivity of FANSe2 increased slightly when allowing more errors in a read. When 7 errors were allowed, the sensitivity of FANSe2 reached 99.99% and 99.0% for 2% and 4% error rate, respectively. Again more than 99% of the reads were mapped using 14-nt seeds, exceeding the sensitivity of all other tested algorithms. Stepping down to 12-nt or lower hardly mapped more reads, thus is practically unnecessary. Note that the error allowance for the whole read cannot be explicitly set when using Bowtie2 and novoalign. Some reads mapped with 7 errors were found in their results, showing that this comparison is fair.

Next, we analyzed the correctness of FANSe2 mapping results using the previously described method [20], plotting the number of reads mapped to wrong locations against the number of reads mapped to correct locations (Figure 4B). In all cases, FANSe2 allowing 6∼7 mismatches mapped more reads correctly to its original position than all other tested algorithms. For the non-masked genome, FANSe2 allowing 7 mismatches mapped 2.2% and 6.9% more reads to their correct positions than Bowtie2 at 2% and 4% error rate, respectively. Meanwhile, FANSe2 mapped 41.0% and 44.5% less reads than Bowtie2 to their wrong positions. Applying increasing mapping quality threshold, only the uniquely mapped reads were kept. Bowtie2 decreased the wrongly mapped reads in the cost of discarding a considerable fraction of mappable reads. At the threshold of mapping quality of 5, FANSe2 mapped 4.5% and 27.8% more uniquely-mapped reads to its correct place than Bowtie2 at 2% and 4% error rate, respectively. BWA performed more robust than Bowtie2 in this test, as increasing the mapping quality threshold do not decrease the number of mapped reads dramatically. However it still mapped less reads than FANSe2. Novoalign mapped comparable number of reads as Bowtie2 and BWA, however it mapped 2∼3 times more mapped to wrong places than Bowtie2 and BWA, and increasing the mapping quality threshold almost do not increase the correctness. Bowtie and SHRiMP2 mapped considerably less reads than the other algorithms, especially at 4% error rate.

As repetitive sequence creates challenges to correct read mapping, masked genome sequence is widely used in major studies to improve the efficiency of sequence alignment (e.g. NCBI BLAST) [21], polymorphism and mutation discovery [28], [29], genome annotation and comparison [30]–[33], *etc*. In clinical diagnosis procedures, such as the non-invasive prenatal diagnosis based on next-generation sequencing, mapping reads to masked human genome is also used as a standard [34]–[37]. Therefore, we also performed read mapping tests using the masked genome sequence provided by UCSC Genome Browser. Compared to the non-masked tests, the sensitivity and correctness of all algorithms increased slightly, because the masked genome sequence is free of repetitive regions. Nevertheless, the scenario remains similar to the non-masked tests: FANSe2 has higher sensitivity while maintaining the correctness.

### Experimental validation of the RNA-seq mapping result by FANSe2

**Figure 5:**
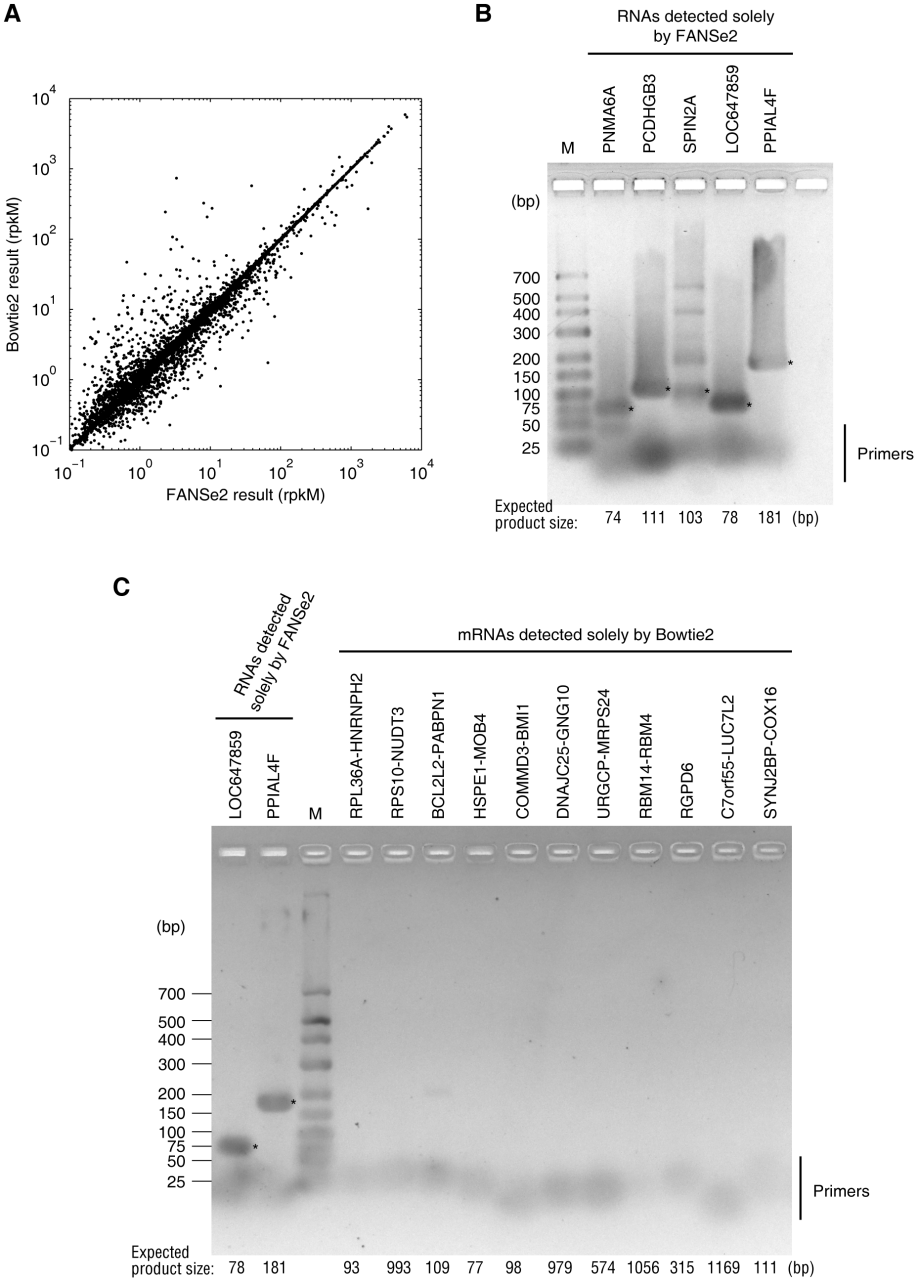
Experimental examination of the results of FANSe2 and Bowtie2. (A) Quantification of mRNA from A549 cells using the mapping results of FANSe2 and Bowtie2. The mRNA sequencing dataset was mapped to the human RefSeq RNA reference sequences and the quantification was performed using the standard rpkM method. (B) RT-PCR validation of mRNAs that were detected by FANSe2 but not by Bowtie2 (see Table 1). 15 µl PCR product were loaded for each lane and resolved on a 3% agarose gel. The bands with the expected product size were marked with stars. The expected product sizes were noted below. (C) RT-PCR validation of mRNAs that were detected solely by Bowtie2 (See Table 2). Two RNAs detected solely by FANSe2 (LOC647859 and PPIAL4F) were loaded as positive control. 7 µl PCR product were loaded for each lane and resolved on a 2.7% agarose gel. The bands with the expected product size were marked with stars. The expected product sizes were noted below. A faint band appeared at ∼200 bp in the lane of BCL2L2-PABPN1 but is quite different than the expected product size.

The robust sensitivity and correctness of FANSe2 maximizes the usage of data in sequencing datasets. This advantage may be more significant when dealing with RNA-seq data that is more error-prone than DNA-seq. In our previous work, we had shown that BWA and BLAT lose mappable reads in low abundance mRNA unproportionally in a prokaryotic system [5]. We next tested FANSe2 and Bowtie2 with our previously reported mRNA-seq dataset (75 nt single-end reads) of human lung cancer cell line A549 [22]. Aiming at quantitative profiling of known mRNAs, we mapped the reads to RefSeq human RNA reference sequence and the splice variants were merged. Previous study showed that mapping to mature mRNA sequence avoided the error of mapping splice junction reads when using genomic sequence as reference and should be preferentially used for RNA-seq, unless novel splice junctions are to be detected [38], [39]. Additionally, protein coding mRNAs consist only a small proportion of the genomic sequence, reducing the computational demand dramatically. Therefore this is an efficient strategy that is widely used by the community [25], [38]–[43]. Genes with less than 10 reads mapped were considered as unreliable quantified genes and removed [23]. We found that the gene expression quantitation of the two algorithms in general coincide for the genes that were identified by both algorithms (Figure 5A).

We next experimentally investigated the genes that were solely identified by FANSe2 (Table 1) or Bowtie2 (Table 2) to check the possible false positives and false negatives. The abundances of the top five RNAs that solely identified by FANSe2 range from 1.47 to 5.77 rpkM (Table 1). They were all validated by RT-PCR with clear bands on the gel at the estimated sizes (Figure 5B). Although the primer specificity of SPIN2A was not high enough so that additional bands appeared in addition to the strongest and expected band, SPIN2A has been detected by microarray in lung adenocarcinoma (Expression Atlas) [44]. In contrast, the abundances of the top 20 RNAs that solely identified by Bowtie2 range from 4.58 to 146.46 rpkM (Table 2). Three genes among them, namely LUZP6, PIGY and SNRPN, are identical in sequence to genes MTPN, PYURF and SNURF, respectively. Therefore, they are undistinguishable to algorithms or RT-PCR and thus excluded from our experimental validation. Indeed, FANSe2 identified MTPN, PYURF and SNURF. Fifteen genes among the top 20 “Bowtie2-only” genes were fusion genes with the abundance of 12.66∼146.46 rpkM, at least one order of magnitude higher than the “FANSe2-only” genes. We verified 10 protein-coding genes among them using RT-PCR, but none of them can be validated (Figure 5C). The coding gene RGPD6 were also failed in the verification (Figure 5C). This experimental verification showed that Bowtie2 results in both false-negatives and false-positives: it fails to identify genes like PCDHGB3, SPIN2A, while erroneously identified the gene RGPD6 and numerous fusion genes that are actually absent in the sample. Meanwhile, a considerable number of reads were assigned to the false-positive identifications by Bowtie2: 11645 reads were mapped to the 11 “Bowtie2-only” genes that were experimentally determined as absent. This may also influence the quantitation of other genes and may be a source that causes the quantitative deviation from FANSe2 (Figure 5A). In contrast, FANSe2 results can be validated by experiments, showing its reliability.

**Table 1:** The top five RefSeq RNAs that are exclusively identified by FANSe2 in A549 mRNA-seq dataset.

		FANSe2		RT-PCR validation		
Gene name	RefSeq-ID	Read count	rpkM	Whole Transcriptome qPCR Primer Database ID	Expected product size (bp)	Validated (Figure 5)
PCDHGB3	NM_018924	105	1.47	PCDHGB3_uc003ljw.2_1_2_2	111	Yes
SPIN2A	NM_019003	60	2.92	PB *	103	Yes
LOC647859	NR_026578	52	5.77	OCLN_uc011cru.1_2_1_2	78	Yes
PNMA6A	NM_032882	45	1.95	PB *	74	Yes
PPIAL4F	NM_001164262	44	3.60	PB *	181	Yes

*PB: primer pair not available in whole transcriptome qPCR primer database. The primers are automatically designed using NCBI-PrimerBLAST. Please refer to Table S2 in File S1 for details.

**Table 2:** The top 20 RefSeq RNAs that are exclusively identified by Bowtie2 in A549 mRNA-seq dataset.

		Bowtie2			RT-PCR validation		
Gene name	RefSeq-ID	Read count	rpkM	Identical to gene **	Whole Transcriptome qPCR Primer Database ID	Expected product size (bp)	Validated (Figure 5)
SENP3-EIF4A1	NR_037926	9640	146.46				
LUZP6	NM_001128619	3264	54.95	MTPN			
RPL36A-HNRNPH2	NM_001199973	2653	65.24		RPL36A-HNRNPH2_uc022cag.1_3_1_2	93	Failed
RPS10-NUDT3	NM_001202470	2240	57.96		PB *	993	Failed
SLMO2-ATP5E	NR_037929	1490	94.87				
BLOC1S5-TXNDC5	NR_037616	1336	26.06				
BCL2L2-PABPN1	NM_001199864	1297	39.82		BCL2L2-PABPN1_uc001wjh.4_2_2_1	109	Failed
HSPE1-MOB4	NM_001202485	1119	17.10		HSPE1-MOB4_uc021vum.1_1_1_2	77	Failed
HNRNPUL2-BSCL2	NR_037946	1092	17.79				
HIF1A-AS2	NR_045406	985	31.40				
ATP6V1G2-DDX39B	NR_037853	969	27.49				
COMMD3-BMI1	NM_001204062	865	16.69		COMMD3-BMI1_uc009xkg.3_4_2_2	98	Failed
DNAJC25-GNG10	NM_004125	675	29.45		PB *	979	Failed
URGCP-MRPS24	NM_001204871	644	50.25		PB *	574	Failed
PIGY	NM_001042616	607	29.27	PYURF			
RBM14-RBM4	NM_001198845	559	22.65		PB *	1056	Failed
SNRPN	NM_022807	559	20.88	SNURF			
RGPD6	NM_001123363	535	4.58		PB *	315	Failed
C7orf55-LUC7L2	NM_001244584	532	12.66		PB *	1169	Failed
SYNJ2BP-COX16	NM_001202547	526	17.59		SYNJ2BP-COX16_uc021rv2m.1_2_1_1	111	Failed

*PB: primer pair not available in whole transcriptome qPCR primer database. The primers are automatically designed using NCBI-PrimerBLAST. Please refer to Table S2 in File S1 for details.

**Identical to gene: two genes have identical sequence and thus are non-distinguishable by the mapping algorithm or RT-PCR.

### Gene expression quantifications by FANSe2 coincide to microarray data better than most other algorithms

**Figure 6:**
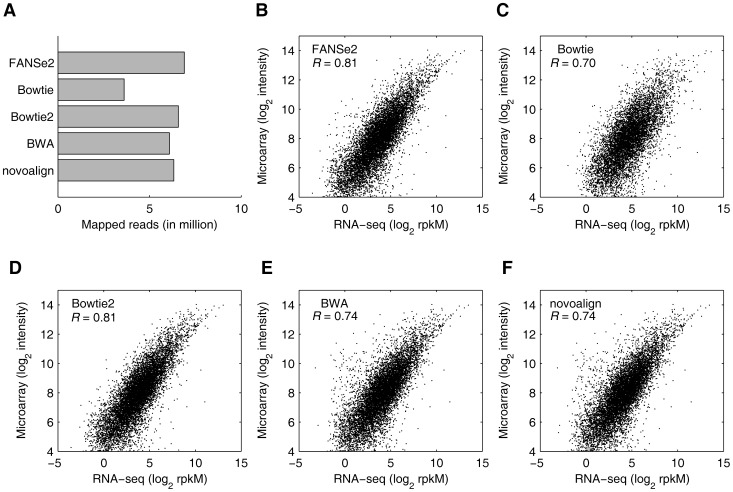
Comparison of the gene expression level calculated by RNA-seq and microarray. (A) The mapped reads to coding genes (NM_*) by FANSe2, Bowtie, Bowtie2, BWA and novoalign. Three mismatches were allowed in the mapping by FANSe2 and BWA. The results of Bowtie were obtained from GEO database GSE21210 and described by Su et al. [25]. (B–F) Correlations of RNA-seq and microarray. Only the reads that mapped to coding genes were taken into consideration to be consistent to the microarray data. The rpkM values of RNA-seq were calculated from the mapping results by FANSe2 (B), Bowtie (C), Bowtie2 (D), BWA (E) and novoalign (F).

Microarray is widely used since decades as a reliable approach to quantify gene expression levels. The hybridization nature of microarray do not need read mapping, providing an experimental reference for mapping-based RNA-seq. We downloaded RNA-seq and microarray data from the same sample (the aristolochic acids treated rat liver sample AA_1) from a previous study by Su et al. [25]. We used FANSe2, Bowtie2, BWA and novoalign to map the same RNA-seq dataset to the same transcriptome reference sequence that was used in [25], and the Bowtie result was taken from Su *et al*.'s report [25]. To be comparable to the microarray used in Su et al.'s study, only the reads mapped to the coding genes were considered. Consistent with the tests above, FANSe2 mapped more reads than the other tested algorithms (Figure 6A). Genes with less than 10 reads mapped were considered as unreliable quantified genes and removed [23]. The FANSe2 result correlates to the microarray data equally good as Bowtie2 (Pearson correlation coefficient *R* = 0.81, Figure 6B and 6D), while the results with other algorithms correlates worse (*R* = 0.70 for Bowtie, *R* = 0.74 for BWA and novoalign, Figure 6C, 6E and 6F). At least in this case on rat, FANSe2 and Bowtie2 provide better consistency to microarray data than other algorithms, facilitating the data integration between different omics platforms. Nevertheless, FANSe2 performed stably also in RNA-seq studies of human cells (Figure 5), showing the advantage of FANSe2 on robustness.

## Discussion

In most of the resequencing and RNA-seq applications, mapping is the bottleneck step in the data processing pipeline [20]. BWT-based algorithms such as Bowtie, BWA and SOAP2 have greatly facilitated the sequencing applications since they are fast enough to perform the mapping on desktop workstations instead of supercomputers (reviewed in [2], [7]). They performed very well for qualitative applications such as DNA resequencing projects, since the loss of mappable reads can be easily compensated by higher sequencing throughput without affecting the results of sequence variation analysis [6]. However the completeness and robustness of the mapping were compromised [5], [6], [12], [16], leading to unproportional loss of mappable reads [5]. This is inacceptable for quantitative applications like RNA-seq. Seed-based algorithms like FANSe offer very high accuracy, quantitativity and robustness, more suitable for quantitative RNA-seq, but usually with much lower speed [5], [6]. To have both advantages, FANSe2 inherited the accuracy of FANSe with largely improved speed due to the iterative step-down strategy based on the statistics of real sequencing datasets. In most sequencing applications, the majority of the reads should be mapped to the reference sequence, and a large fraction of these reads contains very limited number of errors, which can be reliably detected with long seeds at high speed. The accuracy of FANSe2 is ensured by its fully predictable and extremely low theoretical miss rate.

The low correlation between the NGS and microarray platforms in quantitative gene expression studies has been noted in a number of literatures. For miRNA, the Pearson correlation of both techniques reaches only *R* = 0.52∼0.66 [18], [19]. The false negative rates of Illumina NGS platform was as high as 12%, much higher than the microarray platforms (0.97%∼3.1%) [17]. This is also consistent with other studies [18]. Considering the enormous throughput and dynamic range of NGS, this low correlation and high false negative rates is not likely to be caused by the throughput, but by the data processing. As experimentally shown in this study, mapping to RNA reference sequences especially requires the sensitivity and correctness of the mapping algorithm. Algorithms lacking robustness can result in numerous false positives and false negatives in gene identifications and may affect the gene quantification (Figure 5). Also, FANSe2 showed remarkable better consistency to the microarray data than most other algorithms, bridging the gap between the NGS and microarray and leading to better reproducibility and confidence, which is in great demand for the NGS-based studies (reviewed in [45]).

Furthermore, almost all mapping algorithms offer numerous parameters, and small alteration of parameters may lead to significant change of result. This already leads to low reproducibility and low robustness of many next-generation sequencing studies (reviewed in [45]). In contrast, the parameter settings of FANSe2 almost did not affect the sensitivity and correctness (Figure 4B), providing a remarkable simplicity and robustness of usage.

Previous algorithms require more memory for larger reference genomes. For human genome, 3∼14 GB memory is usually required [46]. To reduce the memory consumption when parallelized, some common data, e.g. the reference sequences and the index, need to be shared by multiple CPU cores, increasing the risk of access contention, i.e. simultaneous access of the same data by different CPU cores. This may trigger an unpredictable error or needs additional handling, leading to reduced stability or speed. This problem remains as an open challenge in computational science [47]–[49]. In contrast, FANSe2's memory consumption is almost independent of the reference genome size, since it splits the large reference genome into user-specified segments (Figure 3A). Reducing the segment size can significantly reduce the memory demand without impairing the result, facilitating the parallelization especially in normal office computers. The small and user-adjustable memory consumption also allows parallelization of multiple processes instead of threads, since there is no need to share any common data in the memory, thus eliminating the instability caused by access contention.

Importantly, this merit makes FANSe2 the first algorithm with the feature of flexible, scalable and almost maintenance-free parallelization across multiple computers, efficiently utilizing the computational power of inexpensive office computers and even laptop computers. With just three computers, FANSe2 mapped 608 million reads to human genome within 4.1 hours. This might be the first mapping algorithm that matches the speed of the coming generation of sequencers like Ion Torrent Proton P2 (660 M reads in several hours) running in normal computers. There is no need for expensive, exclusive and maintenance-intensive clusters or workstations.

FANSe2 runs under various operating systems including Windows, providing user-friendly graphical user interfaces, bringing convenience to the biological researchers who are not familiar with computational issues. With simple online video tutorials, everyone knows how to install and use it in 15 minutes. The ability of mapping billions of reads in hours using normal office computers with robust accuracy makes FANSe2 a good candidate to remove the bottleneck of data processing pipeline, leading to much faster, more reliable and quantitative analysis, able to handle the future sequencing applications.

## Supporting Information

File S1 **File S1 includes the following: Table S1.** Six datasets downloaded from DDBJ Sequence Read Archive to analyze the error distribution. **Table S2.** The gene-specific PCR primers for validation of gene identifications. The genes identified solely by Bowtie2 are shaded as gray, and the genes identified solely by FANSe2 are not shaded. **Table S3.** Mapping programs tested in this study. **Table S4.** Test parameters for Figure 4.
(PDF)
