# Supplementary material for: FANSe2: A Robust and Cost-Efficient Alignment Tool for Quantitative Next-Generation Sequencing Applications
Source: PLoS One. 2014 Apr 17;9(4):e94250. doi: 10.1371/journal.pone.0094250 (PMC3990525; doi:10.1371/journal.pone.0094250)
Supplement: File S1 — includes the following: Table S1. Six datasets downloaded from DDBJ Sequence Read Archive to analyze the error distribution. Table S2. The gene-specific PCR primers for validation of gene identifications. The genes identified solely by Bowtie2 are shaded as gray, and the genes identified solely by FANSe2 are not shaded. Table S3. Mapping programs tested in this study. Table S4. Test parameters for Figure 4. (PDF) [file pone.0094250.s001.pdf]

FANSe2: a robust and cost-efficient alignment tool for quantitative next-generation sequencing applications

Chuan-Le Xiao, Zhi-Biao Mai, Xin-Lei Lian, Jia-Yong Zhong, Jing-jie Jin, Qing-Yu He **, Gong Zhang *

Supplementary Materials

Table S1: Six datasets downloaded from DDBJ Sequence Read Archive to analyze the error distribution.

**Accession Number**	**Sample type**	**Sequencer**	**Errors allowed when mapped with FANSe**
ERR039477	*E. coli* genomic DNA	Life Technologies Ion Torrent PGM	9
SRR360459	*E. coli* genomic DNA	Illumina GAIIx	9
SRR057661	*E. coli* genomic DNA	Roche 454 GS FLX	8
SRR028774	Yeast miRNA	Illumina GAIIx	3
SRR363968	Yeast mRNA	Illumina GAIIx	4
SRR034447	Yeast mRNA	Helicos Heliscope	4

Table S2: The gene-specific PCR primers for validation of gene identifications. The genes identified solely by Bowtie2 are shaded as gray, and the genes identified solely by FANSe2 are not shaded.

Gene name	Whole Transcriptome qPCR Primers database ID	Forward primer (5'-3')	Reverse primer (5'-3')	Expected product size (nt)
RPL36A-HNRNPH2	RPL36A-HNRNPH2_uc022cag.1_3_1_2	AGCATTTTGAACTGGGAGGAG	ATCATGGTGATTGTTGGGCT	93
RPS10-NUDT3	PB *	ATGCCTAAGAAGAACCGGATTGC	GCTGCCCACCATGCCTTATT	993
BCL2L2-PABPN1	BCL2L2-PABPN1_uc001wjh.4_2_2_1	GGACAAGTGCAGGAGTGGAT	TCTCCCTGACTCGAGCTTTG	109
HSPE1-MOB4	HSPE1-MOB4_uc021vum.1_1_1_2	ATTGCAAGCAACAGTAGTCGC	CAAAGGATTCATCAGGCCAA	77
COMMD3-BMI1	COMMD3-BMI1_uc009xkg.3_4_2_2	GTTGCAGCATGGAACAATTACA	ACACACATCAGGTGGGGATT	98
DNAJC25-GNG10	PB *	CGCGGGACTGCTACGAG	ACGATGGGGCAGCCTAATG	979
URGCP-MRPS24	PB *	TTGGGAGAAGTAGCCCCAGAA	GTGGAGTCGCACAGGACATT	574
RBM14-RBM4	PB *	GGCTGCGGCGACAAAATGAA	ACACACATCCCACCTCAAGC	1056
RGPD6	PB *	TCCTGCATAGATCTCGTCCTG	TGTATCCTCGGCGACGTC	315
C7orf55-LUC7L2	PB *	AGTCGGTAGTCTGTCCGAC	CTTGCGTTCACGTGACATG	1169
SYNJ2BP-COX16	SYNJ2BP-COX16_uc021rv2m.1_2_1_1	TCGTAATGCAGGCTATGCTGT	CTCTTCACAGCATCATATCGGA	111
PCDHGB3	PCDHGB3_uc003ljw.2_1_2_2	ACGGACTGGCGTTTCTCTC	GGCTTGCAGCATCTCTGTGT	111
SPIN2A	PB *	GCAGAAAAAGGGCAGAGACTAC	CTCGCTGGCCGTCTACCTC	103
LOC647859	OCLN_uc011cru.1_2_1_2	ACAACTGGTGGCGAGTCCT	TTGTTGATCTGAAGTGATAGGTGG	78
PNMA6A	PB *	CGAGTCCCAGGCGACC	CGAAAGCTGAGAGACGCTC	74
PPIAL4F	PB *	GGTGACTTCACACGCCCTAA	TGCCATCCAACCACTCAGTC	181

* PB: primer pair not available in whole transcriptome qPCR primer database. The primers are automatically designed using NCBI-PrimerBLAST.

Table S3: Mapping programs tested in this study.

Mapping tool	Type *	Version	Operating system	Citation
FANSe2	Seed	2.0	Windows 7 64-bit	
FANSe	Seed	1.7.2	Windows 7 64-bit	[1]
Bowtie	BWT	0.12.8	Windows 7 64-bit	[2]
Bowtie2	BWT	2.0.0-beta7	Ubuntu 12.04 64-bit (Linux)	[3]
BWA	BWT	0.6.2	Ubuntu 12.04 64-bit (Linux)	[4]
SHRiMP2	Seed	2.2.3	Ubuntu 12.04 64-bit (Linux)	[5]
Novoalign	Seed	3.02.02	Ubuntu 12.04 64-bit (Linux)	Commercial

* Seed = seed-based algorithm; BWT = Burrows-Wheeler Transform-based algorithm.

Table S4: Test parameters for Figure 4.

Mapping tool	Parameter	Meaning
Bowtie2	--very-sensitive	Recommended most sensitive parameter set
Bowtie	-n 3	The largest mismatch allowed in the mapping to maximize the sensitivity
BWA	-n 5 and –n 7	Allowing 5 or 7 mismatches, comparable to FANSe2 setting.
SHRiMP2	-h 91%	For 75-nt reads, 91% homology is approximately equivalent to 7 mismatches, comparable to FANSe2 setting.
Novoalign	default	
FANSe2	-E5, -E6 and –E7	Allowing 5~7 mismatches

Supplementary References

1. Zhang G, Fedyunin I, Kirchner S, Xiao C, Valleriani A, et al. (2012) FANSe: an accurate algorithm for quantitative mapping of large scale sequencing reads. Nucleic Acids Res 40: e83.
2. Langmead B, Trapnell C, Pop M, Salzberg SL (2009) Ultrafast and memory-efficient alignment of short DNA sequences to the human genome. Genome Biol 10: R25.
3. Langmead B, Salzberg SL (2012) Fast gapped-read alignment with Bowtie 2. Nat Methods 9: 357-359.
4. Li H, Durbin R (2009) Fast and accurate short read alignment with Burrows-Wheeler transform. Bioinformatics 25: 1754-1760.
5. David M, Dzamba M, Lister D, Ilie L, Brudno M (2011) SHRiMP2: sensitive yet practical SHort Read Mapping. Bioinformatics 27: 1011-1012.
